# Transformational nurse leadership attributes in German hospitals pursuing organization-wide change via Magnet® or Pathway® principles: results from a qualitative study

**DOI:** 10.1186/s12913-024-10862-y

**Published:** 2024-04-08

**Authors:** Joan Kleine, Julia Köppen, Carolin Gurisch, Claudia B. Maier

**Affiliations:** 1 Department of Healthcare Management, Technische Universität Berlin, Straße des 17. Juni 135, 10623 Berlin, Germany; 2 School of Public Health, Universität Bielefeld, Universitätsstraße 25, 33615 Bielefeld, Germany; 3 BQS Institute for Quality & Patient Safety GmbH, Wendenstraße 375, 20537 Hamburg, Germany

**Keywords:** Nurse leadership, Transformational leadership, Organization-wide change, German hospitals, Magnet, Pathway

## Abstract

**Background:**

Budget constraints, staff shortages and high workloads pose challenges for German hospitals. Magnet® and Pathway® are concepts for implementing organization-wide change and redesigning work environments. There is limited research on the key elements that characterize nurse leaders driving the implementation of Magnet®/Pathway® principles outside the U.S. We explored the key attributes of nurse leaders driving organization-wide change through Magnet®/Pathway® principles in German hospitals.

**Methods:**

Using a qualitative study design, semi-structured interviews (*n* = 18) were conducted with nurse leaders, managers, and clinicians, in five German hospitals known as having started implementing Magnet® or Pathway® principles. The interviews were recorded and transcribed verbatim. Data were analyzed in Atlas.ti using content analysis. For the analysis, a category system was created using a deductive-inductive approach.

**Results:**

Five leadership attributes and eleven sub-attributes were identified as main themes and sub-themes: Visionary leaders who possess and communicate a strong vision and serve as role models to inspire change. Strategic leaders who focus on strategic planning and securing top management support. Supportive leaders who empower, emphasizing employee motivation, individualized support, and team collaboration. Stamina highlights courage, assertiveness, and resilience in the face of challenges. Finally, agility which addresses a leader’s presence, accessibility, and rapid responsiveness, fostering adaptability.

**Conclusions:**

The study demonstrates leadership attributes explicitly focusing on instigating and driving organization-wide change through Magnet®/Pathway® principles in five German hospitals. The findings suggest a need for comprehensive preparation and ongoing development of nurse leaders aimed at establishing and sustaining a positive hospital work environment.

**Supplementary Information:**

The online version contains supplementary material available at 10.1186/s12913-024-10862-y.

## Background

European hospitals are facing multiple challenges, including economic pressure, cost containment strategies, technological advancements, and shortages of healthcare professionals, which require constant adaptation [1–4]. Particularly concerning is the high burden of mental distress reported by nurses and other healthcare professionals [5, 6]. It is increasingly recognized that the root causes of increased stress and burnout among nurses are linked to the work environment in hospitals and other healthcare settings, that is why hospitals should strive to change their working conditions [7, 8].

The Magnet Recognition Program® (Magnet) and the Pathway to Excellence® Program (Pathway), both originating in the United States (U.S.) and held at the American Nurses Credentialing Center (ANCC), are designed to facilitate organizational-wide change of work environments, enhancing employee well-being, retention, productivity, and patient outcomes [9–11]. These concepts can provide a structured approach for European hospitals to tackle the challenges of the future effectively.

Research on Magnet hospitals, primarily conducted in the U.S., suggests that Magnet can enhance working conditions, job satisfaction, and nurse well-being in hospitals [12–15], while also improving patient outcomes [14–16]. However, findings across studies and outcomes vary. In contrast, the impact of Pathway has received less attention in research. Nevertheless, some studies have indicated that Pathway promotes increased nurse autonomy and decision-making authority, fosters leadership development, improves safety and quality standards, enhances employee well-being, and supports professional growth [17, 18].

As of December 2023, 591 organizations worldwide had Magnet designation [19], and 214 had Pathway designation [20], with the majority in the U.S. Internationally, only 17 hospitals hold Magnet designation [19] and 16 have Pathway designation [20]. While none of them are in Germany, some German hospitals have proactively started implementing Magnet/Pathway principles to drive organization-wide changes aiming at enhancing job satisfaction, attracting and retaining healthcare professionals [21–24]. Hence, the inclusion of Magnet and Pathway principles as comparable case examples of organization-wide change in the current study is justified by the growing proactive adoption of these concepts by some German hospitals.

Magnet and Pathway focus on promoting nurse engagement and supporting professional nursing practice environments. The difference lies in their specific objectives: Magnet emphasizes sustained quality patient care, nursing excellence, and innovations in professional practice, while Pathway highlights creating supportive practice environments that empower and engage staff and is known for having less stringent data requirements [25, 26]. However, both aim to cultivate a culture of nursing care excellence, supported by a transformational leadership style [25], which is the central focus of the present study.

Leadership skills do play a crucial role in successfully promoting organization-wide change [27–29] and has also been shown to have a profound impact on employee stress and emotional well-being [30]. Transformational leadership was identified as one effective leadership style in healthcare settings [29, 31–33]. Transformational leaders motivate employees towards an organizational vision by inspiring and empowering them to continuously develop themselves and addressing their individual needs [34, 35]. The theoretical framework for transformational leadership was first conceptualized in the 1970s, defining it as a relationship between leaders and employees who motivate, empower, and elevate each other’s moral values in pursuit of fulfilling common interests [36]. Further expansion by Bass and Avolio introduced four subcategories that represent the characteristics of transformational leaders: Idealized influence, inspirational motivation, intellectual stimulation, and individualized consideration [34, 35].

Several U.S. studies have analyzed the relationship between transformational leadership and the implementation of Magnet principles [37–39]. In a southern U.S. nonprofit acute care hospital seeking Magnet designation, a survey of 115 staff nurses showed that transformational leadership style was positively associated with nurses’ job satisfaction and satisfaction with promotion opportunities [37]. Magnet hospital CNOs rated their transformational leadership practices highly and reported a strong positive correlation between engagement and leadership practice, with empowering others as the most important practice [38]. A study of clinical nurse leaders, who attended the 2016 Magnet Conference in Orlando, Florida, showed a positive relationship between transformational leadership practices and work engagement, but observed differences in leadership practices and work engagement based on varying levels of education [39].

The majority of research conducted outside of the U.S. has focused on investigating the impact of transformational leadership style within healthcare settings, with no focus on the implementation of Magnet/Pathway [29, 31–33, 37, 40–45]. Several studies found that forms of transformational leadership styles resulted in positive organizational performance, such as improved staff retention, lower turnover, and better quality of care [31–33, 37, 40, 41]. A systematic review including 12 studies from the US, Canada, Saudi Arabia, China, Ethiopia, Italy, and Jordan, showed a positive correlation between transformational leadership and nurses’ job satisfaction in the hospital setting [29].

To date, most of the qualitative studies analyzed transformational leadership style from the nurse leaders’ perspective. A study from Finland used a qualitative design to examine nurses’ leadership skills in leading change [44]. They identified three main roles: First, ‘leading interpersonal relationships’ including competencies of being a team player, coach, and parental figure. Second, ‘leading processes’ including competencies such as organizing, coordinating, and being a conductor based on the organization’s mission. And third, ‘leading a culture’ is defined as advocating values and norms and creating an open, resilient, and evidence-based culture [ibid.]. Another study from 2016 explored senior nurses’ experiences of organization-wide change leadership in three NHS acute hospitals in England through in-depth interviews [43]. The aspect of leadership was frequently discussed in relation to organization-wide change. An effective nurse leader was characterized as a strong, inspiring, and supportive leader with novel and heroic approaches [ibid.]. Weak leaders were those who did not encourage their teams, had poor presence and were unresponsive to the need for change [ibid]. Another qualitative study used a grounded theory approach to examine the processes nursing management uses to promote change on their wards in five hospitals in Japan [45]. According to the interviewees, the change management process led by nurse managers consists of having beliefs and being able to empathize with the nursing staff to achieve common goals [ibid.]. Four characteristics of nurse leaders were reported as indispensable factors for change: having both a micro and macro perspective; respecting their own beliefs and external standards; being proactive; having empathy for nursing staff [ibid.]. A 2020 study conducted in a university hospital in Brazil, examined the challenges of exercising transformational leadership and strategies nurses leaders used to address these challenges include being role models for the team, proactively maintaining dialogue with co-workers, and building empathetic relationships [42].

In German hospitals, a cross-sectional study investigated nursing leadership styles, analyzing the self-assessment of 93 ward managers and the external evaluation of 1,567 employees with the multifactorial leadership questionnaire (MLQ-5X), revealing the presence of transformational leadership practices [46]. The ward management consistently achieved mean values above the scale mean in all dimensions of transformational leadership, both in the self-assessment and in the external evaluation [ibid.]. However, despite the acknowledged existence of transformational leadership practices in nursing within German hospitals, research on the attributes of nurse leaders that support organization-wide change through implementation of Magnet/Pathway principles remains scarce. This study aims to identify beneficial attributes of nurse leaders from German hospitals, shedding light on their role in driving organization-wide change through Magnet/Pathway and advancing the understanding of leadership practices’ impact within the German healthcare systems. Research on this topic is critical to fill a gap in the literature regarding nurse leader attributes that facilitate organization-wide change and can provides insights that could inform nursing leadership development initiatives tailored to the needs of German hospitals seeking Magnet/Pathway designation.

## Methods

### Design and setting

This study was conducted as part of the German Magnet pioneer study, based on a qualitative research design in five pioneer hospitals. Semi-structured interviews were conducted between March and October 2020 with nurse leaders, managers, and clinicians involved in introducing Magnet or Pathway principles in five German hospitals. Inclusion criteria were as follows: (i) hospitals known as pioneers, defined as early adopters of the Magnet or Pathway principles, (ii) having started the implementation on their own initiative prior to 2020. For the purposes of this study, the primary focus was on the leadership attributes of nurses driving organization-wide change using Magnet/Pathway principles. The research protocol was approved by the Ethics Committee of the Charité (No. EA4/185/19). This study used the consolidated qualitative research reporting criteria (COREQ) [47].

The semi-structured interview guide contained a total of nine question with a set of probing questions. Topics covered motivation and rationale for implementing Magnet/Pathway, the identification of facilitators and barriers, of which one question was specifically on the role of leadership. However, interviewees referred to leadership attributes and practices throughout the interview in various instances. All interviewees filled out a short questionnaire on demographic characteristics and information about their role in the hospital, position, and years of work experience.

### Sample recruitment

The purposive sample consisted of 18 persons from the five hospitals. Hospital size ranged between 200 and 2000 beds. Interviewees were nurse leaders, managers, and clinicians who had gained experience with the implementation of Magnet/Pathway. All requested interview partners agreed to be interviewed.

### Data collection and analysis

Interviews were conducted in German and face-to-face by three members of the research team, following the semi-structured interview guide. The interviews lasted between 30 and 135 min. The interviews were anonymized and transcribed verbatim and were coded with ID01-ID18. The analysis of the anonymized transcripts was carried out in a multi-stage procedure based on content analysis, with a content structuring and summarising approach according to Mayring [48].

A deductive-inductive approach was chosen. The transcripts were coded using the data analysis software ATLAS.ti. For the deductive coding a coding guide was developed prior to the analysis based on the five components of the Magnet model [10]. Subsequently, the content of the deductive code leadership was re-analyzed in-depth inductively to answer the research question.

### Quality

Several measures were applied to ensure transparency and quality. This involved investigator triangulation whereby the three researchers were involved in the data collection, analysis, and interpretation of the study [49].

Prior to the interviews, the three researchers conducted pilot interviews among themselves to ensure consistency. The coding of the interviews was performed by the three researchers who also conducted the interviews. Each coder was familiar with all 18 transcripts. After a pilot analysis phase with three interviews which were coded together and discussed at length to achieve high interrater agreement, the transcripts were randomly allocated. In regularly scheduled meetings, the coders reported the interim status and discussed problems or questions regarding the analysis and reviewed sample content of the codes together. In the next step, themes and sub-themes were formed by the researchers and discussed. Examples of quotes from interviewees are provided in the results section to enhance understanding of the interpretation of the results.

## Results

### Interviewee characteristics

The 18 interviewees had a mean age of 48.9 (SD: 10.0) years, seven were female and eleven male. The majority (*n* = 16) had a degree in nursing and two a degree in medicine. Leadership and/or staff responsibilities had 17 interviewees and on average they had five (SD: 2.8) years of experience with implementing Magnet/Pathway (see Table 1).

**Table 1:** Interviewee characteristics

Hospital	H1	H2	H3	H4	H5	Total
Number of participants	4	3	4	4	3	18
Mean age (SD)	48.8 (14.0)	48.0 (11.8)	50.5 (4.8)	49.8 (13.9)	47.0 (8.9)	48.9 (10.0)
Female (%)	2	2	1	0	2	7 (38.9)
Leadership and/or staff responsibility	4	3	4	4	2	17 (94.5)
Academic degree	4	2	3	4	3	16 (88,9)
Mean years of experience:						
• In healthcare (SD)	27.3 (14.1)	27.0 (11.8)	29.0 (8.0)	27.0 (10.6)	29.0 (7.0)	27.8 (9.4)
• With the Magnet/Pathway concept (SD)	4.5 (0.6)	9.3 (3.8)	3.8 (1.7)	2.5 (1.3)	5.7 (0.6)	5.0 (2.8)

SD: Standard deviation; H: Hospital

### Themes

All 18 transcripts were included in the analysis, regardless of whether direct quotes are shown in this study. Five main leadership attributes (subsequently referred to as main themes) driving organization-wide change using the Magnet/Pathway principles were identified: visionary, strategic, supportive, stamina, and agility. The main themes consist of eleven sub-themes (see Table 2).

**Table 2:** Overview of main themes and sub-themes

	Main themes/ attributes	Sub-themes/ attributes
1	Visionary	Having a vision
		Role model
2	Strategic	Strategic planning
		Convincing top management
3	Supportive	Inspiration and motivation
		Individual support of employees
		Team player
4	Stamina	Courage
		Assertiveness
5	Agility	Showing presence
		Accessibility and responsiveness

### Main theme 1: Visionary

Two sub-themes were clustered as the main theme visionary. It emerged that having a vision and acting as a role model were identified as requirement to implement organization-wide change.

#### Having a vision

Most interviewees agreed that leaders need a vision that they carry with conviction and strive to realize with high motivation. Interviewees suggested that a leader who is visionary would inspire employees for the change process. Furthermore, some interviewees mentioned that it is beneficial if leaders communicate well their vision of the future to their employees.

*“With a vision I can inspire people. So, I need something that is strong enough, that really radiates energy, that […] gives people courage.” (ID9)*.

It was seen as essential that the visions should be catchy, based on clinical practice and reflect the needs of clinical practitioners. One of the interviewees also mentioned the involvement of employees in the practical elaboration of the vision.

*“So, I […] presented the vision to my team. […] And we went into working groups on how we can implement it, how we can live it. We have three keywords in there: human, competent, pioneering.” (ID14)*.

#### Role model

The interviewees described the need for people who serve as role models that adhere to their vision, ideals, and values and stand up for them. Leaders acting as role models were identified as being less concerned with fulfilling specific criteria for maintaining a label or certificate but focused on improving the well-being of employees and quality of care. Interviewees explained a role model function of leaders as beneficial when employees could identify with their leaders, as this makes it easier to formulate and accept the vision and values as common goals. One interviewee stressed the importance of the personality of the leader and their manner of communicating with their staff.

*“I would almost reduce it to the personalities that drive the whole thing. So, it always depends on how you transmit something, how do you communicate, how do you deal with your staff.” (ID4)*.

A role model as an inspiration was suggested to increase the motivation of the employees to develop themselves and eventually to work towards the achievement of common goals.

*“For me it was […] exciting to have a nursing director who brought special knowledge and an enthusiasm that I sometimes missed in the nursing field. […] just ahead of the times and […] powerful with motivation and of course that caught me.” (ID2)*.

### Main theme 2: Strategic

Two sub-themes were identified under the main theme strategic: Critical attributes as strategic planning and convincing top management contribute to effective implementation of positive change.

#### Strategic planning

Interviewees mentioned that leadership attributes included the ability to plan strategically to meet the goals of the hospital. For example, one of the interviewees described that the implementation of transformational leadership had been the key to driving further changes in the direction of Magnet.

*“I think that the key component to live the Magnet concept is transformational leadership. […] I first must manage through leadership to keep people and attract new ones. And if that succeeds, […] then I can bring the other components into life […].” (ID9)*.

Some interviewees highlighted the relevance of strategic resource allocation to be able to initiate and sustain the hospital-wide change via Magnet/Pathway principles. This included investing in human resources, e.g. nursing scientists or project coordinators, and in structural development, e.g. data management or digitalization. One interviewee described the strategy of providing budget or other cost-related information for the top management and the board required for Magnet/Pathway implementation.

*“There needs to be at least […] an overarching Magnet project manager or director. […] last year I made an initial rough calculation for the board […] as a template, what it would cost the hospital. […] because that is of course indispensable.” (ID7)*.

#### Convincing top management

Most interviewees addressed the importance of seeking and gaining the support of top management to advance change processes.

*“The first step, […], was to convince the executive director, because the combination is simply necessary to do anything at all.” (ID4)*.

One interviewee underscored the importance of active involvement and dedication from top-level decision makers and management in driving organization-wide change processes.

*“So, I think you definitely need […] - the decision makers, the management - they must commit themselves clearly to it, and they must have a vision in this direction […].” (ID5)*.

### Main theme 3: Supportive

Three sub-themes were clustered under the main theme supportive. To be a supportive leader who fosters an empowering workplace to meet Magnet/Pathway principles, it was described as important to inspire and motivate employees to evolve professionally, support employees individually in developing themselves and their ideas further and cultivate a strong team spirit as a team player to pursue common goals.

#### Inspiration and motivation

Some of the interviewees talked about encouraging and inspiring employees to go beyond themselves. Increasing employees’ self-confidence enabled them to make their own decisions and motivated them to evolve professionally. This increased the motivation to drive positive change within the organization. One of the interviewees further explained that the higher the motivation in the team, the faster positive changes could be implemented.

*“The half of it is […] that you get moving forward and, of course, the more motivated you are, the faster and the better you get moving forward. So, it’s about strengthening motivation and this for the whole team and making sure that you get better professionally.” (ID2)*.

A leader should have a passion for the intended changes to persuade and motivate all employees and should take into account the time component of implementing organization-wide change using the Magnet/Pathway concept.

*“And you have to be passionate about the topic, otherwise the concept won’t work either. That takes time at first. And then the biggest task is to get all the employees on board.” (ID4)*.

#### Individual support of employees

The individual support of employees by leaders was recognized as indispensable when it comes to empowering them. According to the interviewees it was important to create a trusting working environment and to make employees feel that they are supported in developing themselves and their ideas further.

*“[T]he employees must feel something is getting better for me. Managers are standing up for me, they are behind me. That is what is important for employees.” (ID9)*.

However, finding the right support for each employee required individual consultation. Only in this way special circumstances and needs could be considered in a targeted manner. In particular, support for the academic training of bedside nurses is mentioned by interviewees as an example which underscores the significance of management’s role in facilitating and encouraging such endeavors.

*“[…] there was […] a young [male nurse] sitting there […] who says, ‘Yeah, did I get this right, you want us all to have academic degrees?’ And he says, ‘Listen, I’m 35, I have three kids, I can’t afford to give up one euro right now at all.’ And [the CNO] understands, of course, there are priorities. But then you must see how you can support someone like that. […] If someone wants the [bachelor in nursing] […] then there is massive support, especially at the management level.” (ID7)*.

#### Team player

The interviewees shared the idea that inspiration, motivation, and support of staff succeed more effectively when leaders are perceived as team players. According to the interviewees, a strong team spirit strengthens motivation to pursue common goals and helps not to give up. It also supports the well-being of all employees in their daily work.

*“The team spirit is so important because if you motivate a team, if you win a team, and if you, as the leader of such a team, ensure that people enjoy working together and that the day-to-day problems can be sorted out, and if it says at the top and at the front: We are a team, we do this together, and together we are strong, Then you have already won half of everything that can be won.” (ID2)*.

An interviewee at a higher managerial level additionally stated that it is important to always maintain a friendly atmosphere and show appreciation to receive important information about current issues and concerns in the teams. Decisions should not be made alone, but should always be considered with the teams, as they know more about the day-to-day matters of the staff members.

*“We have […] a very, very friendly interaction, because I think that they are not my subordinates, they are my ward managers and they are the most important source of work for me, so to speak. Without them, I wouldn’t need to show up here to work, without my ward managers interacting with their teams, knowing exactly what’s going on here right now […], where’s the tension right now? What’s going well right now, what’s not going well right now?” (ID17)*.

### Main theme 4: Stamina

Two of the sub-themes were clustered as the main theme stamina. It emerged that implementing organization-wide change requires leaders who have strong personalities with courage and assertiveness.

#### Courage

The interviews showed that courage and a willingness to take risks in a context of uncertainty, which requires change, is experienced as a beneficial leader attribute. For the interviewees, courage meant being committed to the community and pursuing a vision and goals and being persistent about them. The step of opting for the implementation of organization-wide change with the Magnet/Pathway is described by some interviewees as a dare and leaders should be prepared for negative effects and to face resistance.

*“I would say: Yes, you can always change something. And I didn’t let myself be discouraged […]. Sometimes people have said that we can’t really implement the Magnet concept here. And for me it was always important which ideas from the concept can be implemented and this I want to implement […], but I don’t let that stop me. […] And of course you also need leaders who are strong enough to say: ‘I know, even if it sounds crazy, we’re going in that direction now’.” (ID9)*.

Nevertheless, especially in times of nursing shortages and high workload, it was important to have a sense of achievement to summon up courage. One of the CNOs interviewed reported that due to a high number of applicants to study nursing in the interviewee’s initial phase at the hospital, the interviewee had gained the courage to believe in the change concept and continued to pursue the interviewee’s vision despite high workloads and poor moods among the nursing staff and continued to pursue their vision.

*“[…] despite reports of work overload, which reached me in droves at that time in my starting phase […], and then I was talking about academic training, actually I almost got a slap in the face, but at the same time [many] applied [to study nursing]. And then that was again the point where I had confidence: ‘You’re sticking to it; you have the courage’.” (ID3)*.

#### Assertiveness

Interviewees agreed that implementing organization-wide change requires a high level of commitment and that leaders should have assertiveness. It was mentioned that it is important to prepare for the long term and to be aware of the length of time required to drive change processes using the Magnet/Pathway concept.

*“I think the concepts themselves are very, very complex and take an incredible amount of time to implement. But it is possible. You just need someone who has stamina and who stands there and says, ‘So, and I want this, and this is the way, and this is my way, and I’m going to follow through.‘” (ID14)*.

A sufficient individual resilience of leaders in hospitals was described as crucial to be able to overcome past failures, learn from them and stay positive. An interviewee described that initial rejection of new intervention plans by employees was initially perceived as a setback, but the leaders remained strong and learned from it.

*“Well, that certainly took its time. [The CNO] came back [from U.S.] to the hospital with lots of new ideas and everyone who hears something new first says: ‘Stop, hold this. […], English words, now we only use English words. What does Magnet mean at all?’ and there was already a lot of distance from the employees […]. But then [the CNO] was able to convince […] at least the mangement level.” (ID4)*.

Convincing top management was also described as an endeavor and requires the attributes of assertiveness and courage. One example is to advocate for one’s employees.

*“And my job as a CNO, of course, is to fight to make sure that my nurse leaders have the resources, from time, to space, to other things, to be able to do good leadership.” (ID9)*.

### Main theme 5: Agility

In the context of this study, agility means the ability to adapt flexibly and quickly to changing tasks, circumstances, and demands as well as fostering a sense of team spirit. Two of the sub-themes were clustered as the main theme agility; showing presence and demonstrate well and fast accessibility and responsiveness.

#### Showing presence

It emerged from the statements of the interviewees that showing presence and direct personal contact of the leaders towards the employees was an important competence to keep up the commitment of the employees for implementing positive change within the hospital.

*“Of course, I’m also in favour of the director of nursing not working at the bedside anymore […], but they should also not forget the direct contact to those who do the frontline work.“(ID2)*.

With personal presence the leader’s appreciation of frontline workers and the interest in their well-being could become more tangible. It would also be a better way to transmit enthusiasm for the overarching, common goal to employees. Likewise, communication lines would be shortened, and employee concerns and needs could be addressed quicker. One of the interviewees shared a key moment when the CNO introduced himself unannounced at a staff meeting, and this sparked a sense of optimism among the employees.

*“And I don’t think there’s ever been anything like this before, where a team meets for a dialogue and all of a sudden the CNO comes in and introduces himself and also says something about his philosophy and how he would like it to be. […] And that was the first jolt, because the employees realized, there’s someone who’s not untouchable, but there’s someone who’s like us.” (ID14)*.

Another interviewee stated that they were expected as a nurse leader to know as many of the employees personally as possible. This was an essential factor for equal interaction between the professional groups.

*“I know a lot of our nurses […], I know all the academic nurses, […] people expect me to take an interest in them. The whole eye-level system means that you really give everyone a name as well, not just a number anymore.” (ID1)*.

One of the interviewees acknowledged that a lack of presence at the frontline work, as well as a lack of interest in the processes by the leader, had negatively impacted the success of implementing change processes.

*“He [CNO] wasn’t on the wards; he didn’t see that as his responsibility either. It was more like; he gives the strategy and then the others are supposed to do the implementation. That just didn’t work out well. […] it was certainly a barrier that the [CNO] had little understanding of the daily operational problems. […] It would still have been easier if the employees had experienced him as being a little closer to the employees.” (ID10)*.

#### Accessibility and responsiveness

In addition, interviewees explained that by showing presence outside of their own office, leaders should also cultivate an “open door”-culture and be available to meet with employees at short notice. This includes accessibility via various communication channels but also quick responsiveness.

*“My door is open all day. I am not the leader who is available once a year for one hour […], but I am permanently there for dialogue, I do have time.” (ID6)*.

*“We do a topical hour with the nursing directorate where they can come and get in touch with science here. [AND] Cappuccino with the nursing directors; where everyone can come without an appointment and also have individual talks […].” (ID3)*.

Another interviewee used a current occurrence to stress the importance of responding to employee requests as soon as possible to keep them motivated.

*“Right before you came in, there was a colleague who asked me […] if he could do a work shadowing. Within three minutes he got his answer […]. What use has a rigid structure, if you let someone like him wait for two weeks and say ‘Well, I was overworked’? You have to address these things.” (ID2)*.

## Discussion

The findings of this study shed light on the attributes of nurse leaders in German hospitals that drive organization-wide change using Magnet/Pathway, which are aimed at improving employee well-being, productivity, and patient outcomes. A key component of the concepts is the practice of transformational leadership [9]. The results highlight five main themes that encompass beneficial leadership attributes: visionary, strategic, supportive, stamina, and agility.

The theme of visionary underscores the significance of visionary leadership in driving organization-wide change towards Magnet/Pathway. Having a clear and compelling vision that is communicated effectively to employees emerged as a key factor. The role of leaders as role models who embody vision and values was also emphasized. This aligns with existing literature on transformational leadership, which emphasizes the importance of idealized influence and inspirational motivation [34, 35]. Leaders who serve as role models and inspire others create a sense of identification and motivation among employees to work towards common goals.

The theme of strategic highlights the role of strategic planning and resource allocation in Magnet/Pathway implementation. Several studies confirmed that strategic thinkers are among the most effective leaders. People with the ability to think strategically are more likely to ensure the sustainable success of an organization and are better able to put existing strategic plans into execution [50–53]. Furthermore, this study emphasizes the importance of leaders advocating for Magnet/Pathway principles to gain the support and commitment of the top management. Convincing the top management was seen as crucial for the success of change efforts.

The supportive theme emphasizes the significance of individualized support for employees’ development and well-being. This includes empowering employees to pursue further education, addressing their unique needs, and fostering a trusting work environment. This study highlights the role of leaders in providing resources and support for academic advancement, which aligns with the Magnet key component on structural empowerment. Furthermore, the importance of team spirit and collaboration was emphasized, echoing the Magnet/Pathway principle of exemplary professional practice and a collaborative work environment. Supportive leaders act as team players and inspire and motivate their staff to perform beyond the norm [38, 54]. They support staff nurses to assessing their own performance, working out their goals and defining their responsibilities [38, 43, 44]. As a result, nurse leaders can promote knowledge building, intrinsic motivation and innovative work behavior among nurses [54].

In the German healthcare system, it is noteworthy that not every nursing director automatically holds a position on the hospital’s executive board, underscoring the importance of engaging top management in organizational change initiatives. Additionally, given that lifelong learning and continuous professional development are not standard practices in nursing in Germany, contrasting with the conditions required for Magnet/Pathway implementation, it highlights the crucial role of CNOs in empowering their staff and fostering a culture of lifelong learning and professional growth.

Results of previous qualitative studies analyzing transformational leadership style from the nurse leaders’ perspective confirm these findings. They include competencies such as a strong, inspiring, and supportive leader with novel approaches. The leaders serve as role models and are able to empathize with nursing staff to achieve common goals [42–45]. By cultivating a visionary outlook and a supportive stance, nurse leaders can effectively foster work engagement, address burnout, and create a motivating environment for healthcare professionals. Importantly, these leadership qualities can be learned and improved over time among nurses through targeted educational interventions, training, mentoring, and hands-on experiences [55–57]. Nevertheless, while some leadership skills can be developed through training and experience, certain innate character traits may provide an inherent advantage in the cultivation of effective leadership [58]. For instance, personality traits such as emotional intelligence, empathy, and authenticity have been linked to leadership effectiveness [ibid.]. Although these traits may not be directly teachable, they may serve as foundational elements that contribute to the development of successful transformational leadership practices.

The alignment between the findings of this study and those from various international contexts raises important implications for both the Magnet/Pathway implementation process and the broader understanding of transformational leadership. The resemblance between the identified leadership attributes and those found in studies conducted in different countries suggests a level of universality in the leadership qualities required for successful organization-wide change, particularly in healthcare settings. Furthermore, the consensus between the leadership attributes identified in this study and those from international research may imply that studies solely focused on transformational leadership can offer relevant insights for organizations pursuing Magnet/Pathway designation.

Given the diversity of leadership approaches across other sectors (including industry), it is important to recognize the contextual relativity of specific leadership traits. While the leadership attributes identified in this study are tailored specifically to nursing within hospitals, they may be relevant in other sectors, but require further evaluations in different contexts. While a leader’s ability to communicate a clear vision and motivate employees is as crucial in the business sector as in healthcare, the best way of communication and motivation may vary.

New insights that expand the understanding of the role of leadership in driving organization-wide change that emerge from this study is the perspective on the themes of stamina and agility in the context of transformational leadership and Magnet/Pathway implementation.

The theme of stamina reflects the perseverance and resilience required for implementing Magnet/Pathway principles. Leaders need courage to navigate uncertainty and take calculated risks. Leaders should want to make changes and push them forward with stamina and assertiveness, and who are not afraid to speak up and ask for support from higher authorities. In the context of transformational leadership, stamina has often been discussed as a leader’s perseverance, determination, and resilience in the face of challenges [34]. However, the findings of this study emphasize a more profound dimension of stamina as the leader’s ability not only to persevere, but also to inspire and sustain momentum during complex, long-term endeavors such as Magnet/Pathway implementation.

The concept of agility as an essential leadership skill has gained attention in recent years and refers to a leader’s ability to adapt quickly to changing circumstances. This aligns with the dynamic nature of healthcare settings, where leaders must respond to changing demands [59]. Interviewees in this study emphasized the positive impact of agile leadership, which enables leaders to respond rapidly, purposefully, and individually to different situations [59–61]. By adapting task structures and difficulty levels to match employees’ needs and commitment, leaders can prevent both under- and over-challenging their team members, thereby facilitating their professional development [59–61]. With the sub-themes showing presence as well as accessibility and responsiveness, it became clear that leaders should be in regular exchange with their employees to be able to react agilely to the requirements and needs [62]. In contrast, the findings confirm that the lack of frontline presence as well as the leader’s lack of interest in the processes negatively impacted the success of Magnet/Pathway implementation. However, in the context of Magnet/Pathway implementation, agility goes beyond flexibility to include a dynamic responsiveness to evolving healthcare challenges. The results show that leaders need to be flexible not only in their decision-making, but also in their accessibility and responsiveness to employees. This reflects a proactive approach that not only supports the implementation of the Magnet/Pathway principles, but also ensures that employees remain engaged and motivated throughout the transformation process.

### Limitations

This study has several limitations. Firstly, the interviews were conducted in only five German hospitals, yet they were the first ones known to have started introducing Magnet or Pathway in Germany. While these hospitals provided valuable insights into the implementation process, the perspectives shared by leaders and staff are not representative of all hospitals in Germany, limiting the generalizability of the findings. Secondly, the majority of interviewees in this study were nurse managers or individuals with leadership responsibilities. As a result, the findings primarily reflect the management and leadership perspective, potentially overlooking the viewpoints and experiences of frontline nurses. It is important to consider a wider range of perspectives to gain a comprehensive understanding of the challenges and opportunities associated with leadership while implementing Magnet/Pathway principles.

## Conclusions

This study provides in-depth insights into the leadership attributes that drives the implementation of organization-wide change through Magnet/Pathway principles in German. It offers guidance for nurse leaders seeking to drive positive organization-wide change and enhance employee well-being.

The interviewees in this study emphasized the importance of leadership competencies such as visionary direction, strategic planning, personalized support, resolute stamina, and adaptive agility. The themes of stamina and agility offer new insights, showcasing the need for courage, assertiveness, and adaptability in leaders driving long-term organization-wide change towards Magnet/Pathway.

Given the vital role of transformational leadership in driving organization-wide change, as well as the fact that transformational leadership skills can be trained, a comprehensive preparation and ongoing development of nurse leaders toward transformational leadership skills may support establishing and sustaining a positive work environment in hospitals.

### Electronic supplementary material

Below is the link to the electronic supplementary material.

Supplementary Material 1

Supplementary Material 2

Supplementary Material 3

## Data Availability

A 32-item checklist for interviews and focus groups (COREQ) and the coding tree are provided as supplementary material. The transcripts used and analyzed during the Magnet pioneer study are available from the corresponding author on reasonable request.

## Abbreviations

ANA: American Nurses Association
ANCC: American Nurses Credentialing Center
CNO: Chief Nursing Officer
COREQ: Consolidated Criteria for Reporting Qualitative
ID: Interviewee Identification
MLQ: Multifactor Leadership Questionnaire
NHS: National Health Service
SD: Standard deviation
U.S.: United Sates of America
