# Supplementary material for: Transformational nurse leadership attributes in German hospitals pursuing organization-wide change via Magnet® or Pathway® principles: results from a qualitative study
Source: BMC Health Serv Res. 2024 Apr 8;24:440. doi: 10.1186/s12913-024-10862-y (PMC11003170; doi:10.1186/s12913-024-10862-y)
Supplement: Supplementary file 1 — Supplementary Material 1 [file 12913_2024_10862_MOESM1_ESM.docx]

**Supporting Information**

Appendix A:

Consolidated criteria for reporting qualitative studies (COREQ): 32-item checklist.

Developed from Tong A, Sainsbury P, Craig J. Consolidated criteria for reporting qualitative research (COREQ): a 32-item checklist for interviews and focus groups. International Journal for Quality in Health Care. 2007. Volume 19, Number 6: pp. 349 – 357.

| **No.** | **Item** | **Guide questions/description** | **Notes** | **Page in manuscript** |
| --- | --- | --- | --- | --- |
| **Domain 1: Research team and reflexivity** | | | |  |
| **Personal Characteristics** | | | |  |
| 1 | Interviewer/facilitator | Which author/s conducted the interview or focus group? | Four interviews were conducted by Claudia Maier, eleven by Julia Köppen and three by Joan Kleine. | 8-9 |
| 2 | Credentials | What were the researcher’s credentials? E.g. PhD, MD | Dr. PH. Claudia Maier  M.Sc. Carolin Gurisch  M.Sc. Joan Kleine  M.Sc. Julia Köppen | Information provided in checklist only |
| 3 | Occupation | What was their occupation at the time of the study? | Claudia Maier was a postdoctoral researcher. Carolin Gurisch, Julia Köppen and Joan Kleine were research associates and doctoral students. | Information provided in checklist only |
| 4 | Gender | Was the researcher male or female? | All researchers are female. | Information provided in checklist only |
| 5 | Experience and training | What experience or training did the researcher have? | Claudia Maier and Julia Köppen have had academic training in qualitative research designs and over five years of experience with qualitative and mixed methods designs.  Carolin Gurisch and Joan Kleine had academic training and experience of qualitative research as well as three years of work experience in this field.  In addition, Joan Kleine has ten years of work experience as intensive care nurse. | Information provided in checklist only |
| **Relationship with participants** | | | |  |
| 6 | Relationship established | Was a relationship established prior to study commencement? | For most interviewees, there was no contact between interviewers and participants prior to the study, except for four persons who were known professionally to individual interviewers prior to the study. | Information provided in checklist only |
| 7 | Participant knowledge of the interviewer | What did the participants know about the researcher? e.g. personal goals, reasons for doing the research | Most participants did not know anything about the researchers apart from their role in the research, except for the four interviewees which knew the interviewers’ interest in Magnet®/Pathway® and improvements of nursing care. | Information provided in checklist only |
| 8 | Interviewer characteristics | What characteristics were reported about the interviewer/facilitator? e.g. Bias, assumptions,  reasons and interests in the research topic | In each interview the interviewers presented themselves, explained their position at the university and their role in the research. | Information provided in checklist only |
| **Domain 2: Study design** | | | |  |
| **Theoretical framework** | | | |  |
| 9 | Methodological orientation and  Theory | What methodological orientation was stated to underpin the study? e.g. grounded theory,  discourse analysis, ethnography, phenomenology, content analysis | Interviews were analyzed according to the content analysis according to Mayring. | 9 |
| **Participant selection** | | | |  |
| 10 | Sampling | How were participants selected? e.g. purposive, convenience, consecutive, snowball | Participants were selected through purposive sampling. In addition, the snowball method was used to identify suitable interview partners in the hospitals fulfilling the applied criteria. | 9 |
| 11 | Method of approach | How were participants approached? e.g. face-to-face, telephone, mail, email | Participants were sent an invitation letter via e-mail. | 9 |
| 12 | Sample size | How many participants were in the study? | The sample consisted of 18 participants. | 9 |
| 13 | Non-participation | How many people refused to participate or dropped out? Reasons? | None. | Information provided in checklist only |
| **Setting** | | | |  |
| 14 | Setting of data collection | Where was the data collected? e.g. home, clinic, workplace | All but one Interview were conducted at the participants´ workplaces. All participants were employed in hospitals. One interview was carried out at the interviewer´s workplace, namely the Technische Universität Berlin. | Information provided in checklist only |
| 15 | Presence of non-participants | Was anyone else present besides the participants and researchers? | No. | 10 |
| 16 | Description of sample | What are the important characteristics of the sample? e.g. demographic data, date | The interviewees were chief nursing officers, nurse and medical managers and nursing staff from five German hospitals. The hospitals were chosen due to their efforts to improve the working environment and quality of care via organization-wide changes through implementing Magnet or Pathway. | 9 |
| **Data collection** | | | |  |
| 17 | Interview guide | Were questions, prompts, guides provided by the authors? Was it pilot tested? | A semi-structured interview guide, which was pilot-tested in advance, was used to conduct the interviews. | 10 |
| 18 | Repeat interviews | Were repeat interviews carried out? If yes, how many? | No. | Information provided in checklist only |
| 19 | Audio/visual recording | Did the research use audio or visual recording to collect the data? | Audio recording with non-internet capable devices was used to collect data. | 9-10 |
| 20 | Field notes | Were field notes made during and/or after the interview or focus group? | Due to audio recording the interviewers did not make notes during the interviews. Lasting impressions were noted after the interviews but not analyzed. | Information provided in checklist only |
| 21 | Duration | What was the duration of the interviews or focus group? | Interviews lasted between 30 and 135 minutes. | 9 |
| 22 | Data saturation | Was data saturation discussed? | Data saturation was discussed and was relevant to determine the sample size. | Information provided in checklist only |
| 23 | Transcripts returned | Were transcripts returned to participants for comment and/or correction? | No. | Information provided in checklist only |
| **Domain 3: analysis and findings** | | | |  |
| **Data analysis** | | | |  |
| 24 | Number of data coders | How many data coders coded the data? | Deductive coding was done by Claudia Maier, Julia Köppen and Joan Kleine. Inductive coding was done by Joan Kleine. | 10 |
| 25 | Description of the coding tree | Did authors provide a description of the coding tree? | The complete coding tree is provided in the appendix B. Overview of main themes and sub-themes is shown in results. | Appendix B  11-26 |
| 26 | Derivation of themes | Were themes identiﬁed in advance or derived from the data? | The topic leadership was identified in advance and coherently included in the interview guide. The identified themes and sub-themes were derived from the data. | 10 |
| 27 | Software | What software, if applicable, was used to manage the data? | Atlas.ti | 10 |
| 28 | Participant checking | Did participants provide feedback on the findings? | No. Transcripts were not given to participants. | Information provided in checklist only |
| **Reporting** | | | |  |
| 29 | Quotations presented | Were participant quotations presented to illustrate the themes/ﬁndings? Was each quotation identiﬁed? e.g. participant number | Quotations are used for presenting the results of the study. Each quotation is marked with a participant number. | 11-26 |
| 30 | Data and ﬁndings consistent | Was there consistency between the data presented and the ﬁndings? | Yes. | 11-26 |
| 31 | Clarity of major themes | Were major themes clearly presented in the ﬁndings? | Five main themes emerged from the interviews, which were named and used for structuring the results of the interviews. Sub-themes were also named and used for subheadings. | 11-26 |
| 32 | Clarity of minor themes | Is there a description of diverse cases or discussion of minor themes? | The content of the interviews was presented per theme/sub-theme, including the description of diverse cases. | 11-26 |
