# Supplementary material for: Transformational nurse leadership attributes in German hospitals pursuing organization-wide change via Magnet® or Pathway® principles: results from a qualitative study
Source: BMC Health Serv Res. 2024 Apr 8;24:440. doi: 10.1186/s12913-024-10862-y (PMC11003170; doi:10.1186/s12913-024-10862-y)
Supplement: Supplementary file 2 — Supplementary Material 2 [file 12913_2024_10862_MOESM2_ESM.docx]

**Supporting Information**

Appendix B:

Coding tree

| **Direction of analysis** | | |
| --- | --- | --- |
| **Deductive code** | **Inductive codes**  **(sub-themes)** | **Clustered codes**  **(main themes)** |
| Leadership | Having a vision | Visionary |
|  | Role model |  |
|  | Strategic planning | Strategic |
|  | Convincing top management |  |
|  | Inspiration and motivation | Supportive |
|  | Individual support of employees |  |
|  | Team player |  |
|  | Courage | Stamina |
|  | Assertiveness |  |
|  | Showing presence | Agility |
|  | Accessibility and responsiveness |  |
