# Supplementary material for: Transformational nurse leadership attributes in German hospitals pursuing organization-wide change via Magnet® or Pathway® principles: results from a qualitative study
Source: BMC Health Serv Res. 2024 Apr 8;24:440. doi: 10.1186/s12913-024-10862-y (PMC11003170; doi:10.1186/s12913-024-10862-y)
Supplement: Supplementary file 3 — Supplementary Material 3 [file 12913_2024_10862_MOESM3_ESM.docx]

**Supporting Information**

Appendix C:

**Semi-structured interview guide: Magnet Pioneer Study**

PART 1: Implementation experience in hospitals

Introduction: You're familiar with the concept of Magnet/Pathway hospitals in the United States. It's becoming increasingly recognized in Germany and Europe.

1. I'm interested in understanding why [your/his/her] hospital decided to implement the Magnet/Pathway concept.
   1. What was the reason or motivation behind this decision?
   2. Who or what initiated the process significantly?
   3. Can you provide a specific example of why your hospital chose to implement Magnet/Pathway?
2. How far along was the implementation process with Magnet/Pathway [in your hospital]?
   1. What was achieved (milestones/successes/was GAP ANALYSIS conducted)?
   2. What was the exact process like (steering committee/staffing, is there a person/multiple persons directly responsible for Magnet (full-time/part-time), specific planning, staff allocation (for how many hours), timeline)?
   3. What was missing until the Magnet/Pathway certification?
3. Has anything changed [in your hospital] as a result of Magnet/Pathway?
   1. What exactly changed?
   2. Provide specific examples (on the unit, management level, patient care, etc.).
   3. How do you assess these changes?
   4. Have any of the changes brought about by Magnet/Pathway persisted to this day?
   5. If so, what? / If not, what were the reasons?
4. How would you evaluate your experiences with the implementation of Magnet/Pathway in your hospital so far? 🡪 (without preempting: e.g., positive/neutral/negative experiences)
   1. Core areas of Magnet: Transformational Leadership; Structural Empowerment; Exemplary Professional Practice; New Knowledge, Innovations, and Improvements; Empirical Outcomes
   2. Pathway elements: Leadership, Shared Decision Making, Safety, Quality, Well-being, Professional Development
   3. Data collection/what data is collected, how is it selected (which criteria), how does it work?
   4. Benchmarking (within Germany/internationally)
   5. Financing (ANCC fees)
   6. Authorship/logo
   7. Could the Magnet/Pathway concept be effectively applied to your hospital (culturally/special features of the German healthcare system)
5. What was helping and/or hindering factors in the implementation process?
   1. What specifically positively supported or accelerated the implementation? (Individuals, leadership, processes, structures, etc.)
   2. Were there obstacles, and if so, what were they? How were they addressed?

PART 2: Example: Integration of University-educated nurses (Bachelor/Master)

1. I'm now interested in a specific example of Magnet/Pathway concept implementation:

**Magnet:** the integration of university-educated nurses (with at least a bachelor's degree). The Magnet Manual stipulates the formulation of goals to increase the proportion of university-educated nursing personnel. How have you implemented this [or how do you plan to implement this]?

**Pathway**: there is a point "Professional Development”. Were there any developments related to the integration of university-educated nurses in this context?

1. How is success measured/monitoring conducted? What was set as the target value (percent)?
2. What measures are taken for the education, recruitment of nurses with bachelor's degrees?
3. What measures are taken for integration into nursing practice? d. What sets your hospital apart from others in dealing with nurses with bachelor's degrees?

PART 3: Transfer of the U.S. Model to Germany

So far, we've discussed your experiences in implementing the Magnet/Pathway concept at your hospital.

1. To what extent do you think the U.S. Magnet/Pathway concept is applicable to other hospitals in Germany?
   1. Would anything need to be changed for the transfer to Germany, if so, what?
      1. Core areas of Magnet: Transformational Leadership; Structural Empowerment; Exemplary Professional Practice; New Knowledge, Innovations, and Improvements; Empirical Outcomes
      2. Pathway elements: Leadership, Shared Decision Making, Safety, Quality, Well-being, Professional Development b. Specifically, the manual.
      3. Data collection/what data is collected, how is it selected (which criteria), how does it work?
      4. Benchmarking (within Germany/internationally)?
      5. Financing (ANCC fees)
      6. Authorship, logo
      7. Cultural/language/special features of the German healthcare system.
2. What could be the reasons why only a few hospitals in Germany have opted for Magnet/Pathway?
   1. What factors do you think have hindered the implementation in Germany?
   2. Would implementation on a nationwide scale be conceivable for Germany?
   3. If not, why?
   4. If yes, what would need to change?
3. In your opinion, what factors would promote or accelerate implementation Magnet/Pathway in Germany?
   1. What needs to change (structures, processes, laws) that have previously hindered implementation?
   2. What exactly do you propose?
